# Frequency and determinants of timely arrival among patients of acute myocardial infarction at a public sector tertiary care hospital in Karachi

**DOI:** 10.12669/pjms.36.5.2104

**Published:** 2020

**Authors:** Faryal Akber Jalbani, Shiraz Shaikh, Subhani Fatima

**Affiliations:** 1 Dr. Faryal Akber Jalbani, Research Department, National Institute of Cardiovascular Diseases, Karachi, Pakistan; 2 Dr. Shiraz Shaikh, APPNA Institute of Public Health, Jinnah Sind Medical University Karachi, Pakistan; 3 Dr. Subhani Fatima, Research Department, National Institute of Cardiovascular Diseases, Karachi, Pakistan

**Keywords:** Cardiac emergency, Timely Treatment

## Abstract

**Objective:**

To determine the time from onset of symptoms to start of fibrinolysis and treatment in acute ST elevated myocardial infarction patients and identify the factors which cause delay in treatment.

**Methods:**

A cross sectional study was conducted at National Institute of Cardiovascular Diseases, Karachi on 360 conveniently selected patients of ST elevated myocardial infarction from July to September in the year 2017. Structured questionnaire was used to obtain detailed information on socio-demographics, factors which cause delay and timing of onset of symptoms to arrival of patient in emergency ward.

**Results:**

Overall, the total average time from the start of symptoms to initiation of treatment was 119.85+-63.32 minutes.. Only 5.1% patient reached within one hour while 57.7% reached within two hours. Old age group of 60 and above was positively associated with timely arrival (OR=2.75, 95% CI 1.33-5.68, p=0.006). Significant positive association of using personal car as mode of transport to reach the hospital (OR=5.25, 95% CI 2.94-9.35, p<0.001) was also found as compared to using ambulance. Distance from facility was suggestive of negative association in the model but was statistically insignificant.

**Conclusion:**

According to the findings of this study, more than one third of patients reached the hospital within two hours of initiation of symptoms while only 5.1% reached within one hour. The delay was mostly pre-hospital attributed to arranging transport, stay at first medical contact and time taken from first medical contact to the hospital.

## INTRODUCTION

The World Health Organization estimated in 2016 that ischemic heart disease is the leading cause of deaths worldwide accounting for almost 16% of all deaths.^1^ It is suggested that the patients who present with the signs and symptoms of ST elevation myocardial infarction (STEMI) within 12 hours of symptom onset, and with persistent ST-segment elevation should be treated with early mechanical percutaneous intervention (PCI) or pharmacological reperfusion.^2^ Timing of treatment is the most crucial factor for AMI patients and evidence suggests that Fibrinolysis and reperfusion are more effective if performed within two hours of onset of symptoms.^3^ This is strongly supported by clinical studies confirming the important relationship between achieving prompt ante-grade coronary flow of the infarct artery and improved clinical outcomes.^4^,^5^ Patients not treated with reperfusion therapy on time have significantly higher mortality in the range of 15–25%.^6^ The risk of 1-year mortality is also increased by 7.5% for each 30-minute delay in treatment.^3^ However, >50% patients with AMI report delay in seeking medical care by more than two hours, and >25% patients with AMI delay seeking care by more than six hours.^7^,^8^

Importance of time in patients with acute myocardial infarction has long been recognized. Studies reinforce the need for timely therapy and emphasize the importance of understanding region-specific reperfusion delays. There is dearth of evidence which elaborate the factors which cause this delay in Pakistani population. The objectives of this study were to determine the time from onset of symptoms to start of fibrinolysis and treatment in acute ST elevation myocardial infarction patients and identify the factors which cause delay in provision of fibrinolysis and reperfusion treatment.

## METHODS

### Study Design, Setting and Population

This was a descriptive cross sectional study. It was conducted at the National Institute of Cardiovascular Diseases, Karachi from July to September in the year 2017. The data was obtained from newly admitted Patient of age >18 years of either gender with Positive clinical and ECG findings of myocardial infarction consenting to participate in the study. ST elevation myocardial infarction was defined as having ST segment elevation in two contiguous precordial leads in men and 1.5mm for women and 0>1mm in other leads (2 contiguous).

### Sample Size and Sampling

Sample size was calculated on the basis of statistics which were taken from pervious study in which 69% patients had delay in seeking treatment.^9^ At confidence level of 95% and margin of error of 5% and non-response adjustment of 10%, sample size calculated came out to be 360. Non-Probability Convenience sampling was done. The patients were interviewed after they were in a completely stable position.

### Data Collection Procedure

Structured questionnaire was used to obtain detailed information on socio-demographics, factors which cause delay (Time of incident, place of incident, distance from facility, type of transport used) and timing of onset of symptoms to arrival of patient in emergency ward of National Institute of Cardiovascular Diseases. The questionnaire was developed after review of literature on similar previous studies. Data was collected by medical students who were trained on data collection procedure. After training, the questionnaire was piloted and revised according to the information and feedback provided by the data collectors. Those patients who fulfilled the inclusion criteria of the study were asked for written consent. The Forms were checked daily for completeness and incomplete forms were excluded. Data was entered twice in SPSS in the form of numeric codes assigned to the variables. The data was also verified by cross validating it with 10 randomly picked forms from the hard copies.

### Statistical Analysis

Data was analyzed using software of Statistical package of Social Sciences (SPSS version 21). Mean + SD was calculated for continuous variables and results of categorical variables were expressed in frequencies and proportions. Average time to arrange transport, reach first medical contact, stay at first medical contact, reach NICVD and initiation of treatment after reaching NICVD was reported as mean and standard deviation. Unadjusted and Adjusted Relationship between independent variables mentioned above and outcome variable i.e. Timely start of treatment (<120 minutes) was assessed using logistic regression analysis in which odds ratios with 95% confidence intervals were computed. P-value of less than 0.05 was considered significant. Patients reporting with AMI for medical care within 2 hours were considered to have arrived timely.^7^

### Ethical Consideration

The ethical approval of the study was taken from Institutional Review Board of Jinnah Sindh Medical University on February 13, 2016 (Reference No: JSMU/IRB/2015/-27). Permission for the data collection was taken from administration of NICVD. Informed written consent wassought from all participating patients. The respondents were assured of the confidentiality of the information that they provided.

## RESULTS

Descriptive statistisc of socio demographic variables are provided is in Table-I. The mean age of patients was 55.29 +/- 6.973. Almost one fourth (25.1%) were in the young age group of 35-50 years and another one fourth (25.6%) in the old age group of 60 and above. The remaining half (49.3%) patients were between 51-59 years of age. Males (86.5 %) outnumbered the females (13.5%).

**Table-I:** Socio-demographic characteristics of the study participants (n=360).

	Percentage %	(Frequency)
***Age***		
35-50	25.1	(93)
51-59	49.3	(183)
60 and above	25.6	(95)
***Gender***		
Male	86.3	321
female	13.2	49
***Occupation***		
Self employed	20.2	(75)
Govt. employed	7.5	(28)
business	20.8	(77)
Labor	8.9	(33)
retired	16.2	(60)
Private employs	17.8	(66)
House wife	8.6	(32)
***Education***		
Primary	30.7	114
Intermediate	32.9	122
Graduate	36.4	135

Average time from onset of symptoms to initiation of treatment is shown in Table-II. The average time to arrange transport was 9.35 +-7.74 minutes. If applicable, the average time to reach first medical contact was 14.76 +-7.38 minutes and average stay there was 43.98+-33.94 minutes. The average time taken from FMC to NICVD was 35.34+-11.45 minutes and average time of initiation of treatment at NICVD was 18.84+-9.85 minutes. Overall, the total average time from the start of symptoms to initiation of treatment at NICVD was 119.85+-63.32.

**Table-II:** Average time from onset of symptoms to initiation of treatment (n=360).

	Mean	SD
Average Time to arrange transport	9.3557	7.74495
Average Time to reach first medical contact	14.7668	7.3868
Average Stay of duration at FMC	43.98	33.948
Average Time taken from FMC to NICVD	35.3499	11.45268
Average Time at NICVD treatment	18.8484	9.85570
Total time from first symptom to initiation of treatment at NICVD	119.857	63.32502

The Multivariate Logistic Regression Analysis on association of Socio-demographic characteristics, co-morbids and incident characteristics with timely arrival (<120 minutes) of patients are shown in Table-III. Taking young age of 35-50 years as reference, age group of 60 and above was positively associated with timely arrival. (OR=2.75, 95% CI 1.33-5.68, p=0.006). Female gender was suggestive of positive association but was statistically insignificant. Angina and hypertension were suggestive of positive association but were statistically insignificant while Diabetes (OR=2.26, 95% CI 1.32-3.87, p=0.003) showed significant positive association with the outcome. Significant positive association of using personal car as mode of transport to reach the hospital (OR=5.25, 95% CI 2.94-9.35, p<0.001) in comparison to using ambulance was observed.

**Table-III:** Relationship of socio demographic characteristics, co-morbids and incident characteristics with timely arrival of patients (n=360).

	OR (95% CI)	P-value
***Age***		
35 to 50	1.00	
50-59	1.53 (0.84-2.81)	0.162
Above 60	2.75 (1.33-5.68)	0.006
***Gender***		
Male	1.00	
Female	1.72 (0.70-2.44)	0.163
***Education***		
Up to Primary	1.00	
Intermediate	0.58 (0.30-1.10)	0.099
Graduate	1.16 (0.61-2.19)	0.633
Angina	1.32 (0.76-2.30)	.310
Hypertension	1.23 (0.45-3.39)	0.678
Diabetes	2.26 (1.32-3.87)	0.003
***Transport***		
Ambulance	1.00	
Personal car	5.25 (2.94-9.35)	<0.001
Other	4.95 (2.24-10.97)	<0.001
***Time of incidence***		
Evening (17:00-20:00 hrs)	1.00	
Morning (6:00-11:59 hrs	1.66 (0.80-3.40)	0.167
Night (20:01-5:59 hrs)	1.57 (0.69-3.57)	0.276
Afternoon (12:00-16:49 hrs)	1.28 (0.53-3.07)	0.571
Went to FMC	0.02 (0.003-0.20)	0.001
***Place of Incidence***		
Home	1.00	
Other	1.83 (0.88-3.78)	0.101
***Distance from NICVD***		
Up-to 5 km	1.00	
6 to 15 km	0.72 (0.39-1.31)	0.288
More than 15 km	0.73 (0.37-1.43)	0.366
Patient Was Conscious	5.24 (2.37-11.62)	<0.001
Availability of Family Member	0.82 (1.07-2.59)	0.190

Distance from facility was suggestive of negative association in the model but was statistically insignificant. Going to First Medical Contact before the hospital (OR=0.02, 95% CI 0.003-0.20, p=0.001) was significantly negatively associated with the outcome.

## DISCUSSION

The results from this study highlight the delay in thrombolytic treatment and factors associated with it. In our study average time from onset of symptoms to start of therapy was 119.85+/-63.32 minutes. Longer average times of more than two hours have been reported in studies conducted in USA, Greece and Tehran.^9^-^11^ In a more recent study from Brazil mean time between symptom onset and arrival at the PCI hospital ranged from 9 to 24 hours.^12^ The delay was predominantly pre-hospital as average time from reaching the hospital to initiation of treatment was only 18.84 minutes. This finding is similar to the findings of a study in Pakistan in which treatment of acute coronary syndrome was on average initiated within ten minutes after reaching the hospital.^13^

In this study, only 5.1% patients reached the hospital from onset of symptoms within one hour hours whereas 57.7% reached between one to two hours while 37.2% reached after more than two hours. This is consistent with a recently published study in Pakistan which reported 8.2% reaching the hospital within one hour.^14^ Almost two thirds (62.8%) of patients reached within two hours which is considered a cutoff for timely initiation of treatment. This is better than some old studies conducted in USA between 2001 and 2008 which showed only 41% to 45% patients reaching within two hours.^7^,^8^,^15^ Similarly, study conducted in Tehran on 513 patients in 2012 showed only 19% patient reaching within two hours.^9^ However, more recent studies from the developed countries show much less reaching times as compared to this study. A study done on 3219 patients in 2010 in USA show that 44% patients reached the hospital within 30 minutes of symptoms.^16^ Similarly, another study done in Turkey in 2008 showed that 31% arrived within 60 minutes as compared to only 5% in this study.^17^ It can be said that keeping in view the current standard of >2 hours considered as delayed arrival, 37.2% reaching late is a huge number. Therefore, it is important to understand what determines the timely and delayed arrival and an attempt has been made to analyze the possible factors that could have influenced the timely arrival.

Our results show that the younger patients are more likely to receive treatment late than the elderly patient. This could be due to the fact that older patients over the period of time are more knowledgeable about the symptoms and are more cautious and aware about the importance of reaching early. While literature supports the positive influence of age on reaching timely, ageing in excess of >75 may reverse the trend and cause delayed arrival due to physical limitations of too old an age revealed in a couple of studies done in USA.^8^,^16^

Gender did not show any significant difference in overall duration of arrival in this study, however, positive trends were observed for females to arrive more timely as compared to males. This could be due to the fact that females are usually at home and can manage to arrange transport quickly as compared to males who can be at awkward places at time of onset of symptoms. These results are similar to a previous study done in Tehran in 2012 which has reported that females reach earlier than males.^9^ On the contrary, studies from the developed world report that females reach later than men or take equal time as males because male female working ratios are almost equal^8^-^10^. Being educated has no relationship with timely arrival in this study. On the contrary, the study from Turkey reports that people who have education level more than nine years reach significantly earlier in cardiac emergencies.^17^ The reason for this discrepancy could possibly be low role of education in influencing behaviors in Pakistani society.

Existing Co morbid conditions like diabetes, hypertension and angina can also have positive effect of being more careful, cautious and alert. In this study also, presence of these co-morbids was also positively associated with timely arrival. It has been previously reported that lack of knowledge of symptoms of heart attack are significantly associated with pre-hospital delay^18^. On the contrary, these co-morbids can also either mask the symptoms or cause more severe symptoms which may result in delayed arrival as reported in a few studies0.^8^-^10^

Patient who had personal car reached much earlier as compared to those who had to arrange ambulance to reach the hospital. Due to long waiting times and non-availability of pre-hospital emergency care in ambulances, it is not surprising that most people prefer other modes of transport to reach the hospitals. This is also reflected in a study done in Africa in 2016 that showed that the majority (69%) of patients were driven to hospital in a private vehicle while only 16% used an ambulance.^19^ Although statistically insignificant, Patients arriving in the morning and night time were more likely to reach on time than those coming in the evening which can be explained as high rush of traffic in the evening hours. This is consistent with a study in USA which also reports significantly longer delays in patients with onset of symptoms in the afternoon or evening^8^. Similarly, distance was negatively associated with reaching timely to hospital. Understandably, people who went to first medical contact before reaching the emergency care took significantly longer time to reach. This is suggestive of low awareness of people in general about where to go in cardiac emergencies. This also reflects limited number of cardiac emergency facilities available in a highly populated big city of Karachi.

### Study Limitations

The study has attempted to estimate the average time taken to reach the hospital by emergency patients of acute myocardial infarction and identify the determinants of timely arrival in the major cardiac facility of the biggest city of Pakistan. However, this study represents the experience of a single tertiary center due to time and resource constraints. In a way, the study site represents a huge number of patients coming from all parts of the city, it will be worthwhile exploring the dynamics of urban, semi-urban and rural dimensions of cardiac emergencies in the future. Second, the time intervals were measured on the basis of the information provided by the patient or his or her relatives. Furthermore, information on initial symptoms experienced was incomplete; therefore, it could not be analyzed. However, there is no other way of getting the information regarding the pre-hospital delay except determining the hospital delay using the hospital admission records. Last, this study does not attempt to correlate the average time with survival of the patient due to low sample size for determining mortality. In future, such a study may be designed to estimate the safety period in which can be linked to high survival of patients.

## CONCLUSION

According to the findings of this study, more than one third of patients reached the hospital within two hours of initiation of symptoms while only 5.1% reached within one hour. The delay was mostly pre-hospital attributed to arranging transport, stay at first medical contact and time taken from first medical contact to the hospital.

### Study Recommendations

Based on the findings of the study, we propose a few recommendations. First, education had no significant influence on reaching timely, as a result, people particularly without co-morbids were more likely to reach late. There is a need to provide mass awareness on early identification of symptoms and where to report urgently when these symptoms are experienced. Second, most of delays were due to going to first medical contact from where the patient had to be referred to emergency care. There is a need of increasing the number of emergency coronary care units and raising public awareness on where to go for emergency coronary care.

Third, it was found that people coming by ambulances reached significantly late and there was preference for using personal transport. Therefore, it is high time that response times of ambulances are reduced by enhancing the networks and all services are equipped with standard pre-hospital emergency care including equipment and trained emergency technicians. There is also a need of better co-ordination of ambulances with hospital emergency care units where information related to patient is constantly shared in emergency situation. Lastly, delay was also caused by evening traffic hours, therefore, ambulance friendly rules and laws need to be promoted and implemented for facilitating the right of way for ambulances especially during rush hours.

### Authors’ Contributions

**FA and SS** conceived the idea, developed the proposal, conducted the trainings of data collectors, supervised and analyzed the data and wrote the discussion. Both the authors are responsible and accountable for the accuracy or integrity of the work.

**SF** helped in literature search, data entry and analysis. She also developed the data collection strategy.
